# Hematological Parameters in Severe Complicated Plasmodium falciparum Malaria among Adults in Aden

**DOI:** 10.4274/Tjh.2012.0086

**Published:** 2013-12-05

**Authors:** Sawsan Bakhubaira

**Affiliations:** 1 Aden University, Faculty of Medicine and Health Sciences, Department of Hematology Oncology, Aden, Yemen

**Keywords:** Complicated, Severe, Malaria, Thrombocytopenia

## Abstract

**Objective:** To study some hematological parameters in adult patients with complicated severe malaria and their relations to clinical outcome.

**Materials and Methods:** This was a prospective study, including 77 patients from Aden Governorate with complicated severe malaria over the course of 2 years (2010-2011).

**Results: **The common form of severe malaria in Aden was cerebral malaria (25.9%), followed by renal failure (18.2%), severe anemia (16.9%), and hepatitis (14.3%), with a case fatality rate of 7.8%. Hemoglobin concentration was significantly different among the various complications of severe malaria, while platelet and white blood cell counts did not show such differences. The mean age was older among patients that died. Hematological parameters did not significantly differ among dead or surviving patients. Thrombocytopenia was seen in 42.9% of patients and 18.2% of these had platelet counts of <50.0x109/L. However, none of them developed bleeding.

**Conclusion:** This study concluded that hematological changes are common complications encountered in severe malaria, but they are not related to the clinical outcome.

**Conflict of interest:**None declared.

## Abstract (tr)

**Amaç:** Komplike ağır sıtmalı hastalarda bazı hematolojik parametrelerin klinik sonuç ile ilişkisinin incelenmesi.

**Gereç ve Yöntemler:** Aden vilayetinde iki yıllık sürede (2010-2012) tanı alan 77 komplike ağır sıtma hastasını içine alan prospektif bir çalışma yapıldı.

**Bulgular:** Ağır sıtmanın çeşitli komplikasyonlarında hemoglobin konsantrasyonları önemli ölçüde farklı idi, bununla birlikte trombosit ve lökosit sayıları bu farklılığı göstermedi. Yaş ortalaması ölen hastalar arasında büyüktü. Hematolojik parametreler, ölen ya da hayatta kalan hastalar arasında önemli derecede farklı değildi. Trombositopeni oranı %42,9 idi ve bunların %18,2’sinde sayı 50,0 x109/L’den küçüktü. Ancak hiçbirinde kanama gelişmedi.

**Sonuç:** Bu çalışma ile, hematolojik değişikliklerin ağır sıtmada sık karşılaşılan komplikasyonlar olduğu fakat klinik sonuç ile ilişkili olmadığı sonucuna varılmıştır.

## INTRODUCTION

Malaria is an important cause of death and illness, especially in tropical countries [1]. The most severe forms of and deaths from malaria are caused by Plasmodium falciparum, with other species rarely producing serious complications, debilitating relapses, and even death [2]. Major complications of severe malaria can develop rapidly and progress to death within hours or days [3]. These include cerebral malaria, pulmonary edema, acute renal failure, severe anemia, and/or bleeding. Acidosis and hypoglycemia are the most common metabolic complications [1,2,3,4]. 

The World Health Organization (WHO) established criteria for severe malaria that assisted clinical and epidemiological studies. This project was begun in 1990 [4] and was then revised in 2000 to include other clinical manifestations and laboratory values that portend a poor prognosis based on clinical experience in semi-immune patients [3]. 

In Yemen, malaria remains a significant health problem, with Plasmodium falciparum as the predominant species that is responsible for 90% of malaria cases [5]. Different studies were conducted on severe malaria, such as that of Al-Taiar et al. [6] on pediatric patients, which showed severe malaria constituting 17% of pediatric hospital admissions. In the same study, the main presentation of severe malaria was respiratory distress (40%), followed by severe anemia (37%) and cerebral malaria (8%). Another study conducted by Al-Mekhlafi et al. [7] reported that severe malaria puts a high burden on health services in Yemen, and they found a high prevalence of severe malaria among children younger than 5 years old. 

The previous studies on severe malaria in Yemen did not focus on the hematological parameters of complicated severe malaria in adults; therefore, this study was conducted to evaluate the main hematological parameters in adult patients with complicated severe malaria in Aden, where a large central referral hospital and a branch of the National Center of Public Health Laboratories (NCPHL) are present. 

## MATERIALS AND METHODS

This was a prospective study that included 77 patients from Aden Governorate with complicated severe malaria admitted to Al-Gamhouria Modern General Hospital (Aden, Yemen) and treated at the NCPHL in Aden during 2 years (2010-2011). Patients were selected according to the following criteria: adult Yemeni patients with severe malaria (aged 18 years or more), confirmed to have Plasmodium falciparum malaria parasites in the peripheral blood or bone marrow, and presenting with severe malaria as according to the WHO definition [3]. Severe malaria patients were followed within the hospital until death or discharge (not including discharge against medical advice).

Exclusion criteria included severe malaria patients of <18 years of age, severe malaria patients who were not residents of Aden (because they usually refuse to stay in the hospital after initial improvement), and severe malaria patients who absconded or were discharged against medical advice. 

The hematological data were collected from the NCPHL in Aden. The clinical data were obtained directly from patients or relatives as well as from physicians in the intensive care unit of the internal medical department of the hospital, where complicated severe malaria patients are admitted. 

In the NCPHL, malaria is diagnosed by thick and thin blood films. These films were stained with Giemsa for the detection and characterization of Plasmodium falciparum malaria and the parasite load was determined for each film. 

Diagnosis of severe malaria was established according to the WHO criteria published in 2000 [3]: 

- Severe anemia was defined as normocytic normochromic anemia with hematocrit of <15% or hemoglobin of <5 g/dL in the presence of parasitemia at >1000/µL. In the case of microcytic/hypochromic anemia, iron deficiency, thalassemia, and hemoglobinopathy were ruled out. 

- Cerebral malaria was defined as unrousable coma not attributable to any other cause, with a Glasgow Coma Scale score of ≤9, or coma that persisted for at least 30 minutes after a generalized convulsion. 

- Renal failure was defined as urine output of <400 mL/24 h and serum creatinine of >3.0 mg/dL despite adequate volume repletion. 

- Circulatory collapse (algid malaria) was defined as systolic blood pressure of <70 mmHg with cold, clammy skin or a core-skin temperature difference of >10 °C. 

- Black-water fever was defined as the passage of dark red, brown, or black urine secondary to massive intravascular hemolysis and resulting hemoglobinuria. 

- Hepatitis was defined as the presence of clinically detected jaundice with serum bilirubin concentration of >3 mg/dL and ultrasonographic evidence of enlarged, inflamed liver with elevated liver enzymes. 

- Hypoglycemia was defined as plasma glucose level of <40 mg/dL. 

- Prostration and weakness was defined as a patient who could not sit or walk with no obvious neurological explanations. 

**Statistical Analysis**

The collected data were analyzed with SPSS 18. Quantitative variables were presented as mean values, standard deviations, and ranges. Results were tested by the Mann–Whitney or Student t-test with a 95% level of significance and p≤0.05 was considered statistically significant. 

**Ethical Considerations**

This study was approved by the ethics committee of the hospital as well as by the ethics committee of the NCPHL in Aden. During data collection from patients or relatives, verbal consent was obtained, and names and personal data were completely secured and transferred to codes to keep patients’ identities private. 

## RESULTS

A total of 88 patients were diagnosed with complicated severe malaria, and 11 of these were excluded due to leaving the hospital early against medical advice. The remaining 77 patients were followed until discharge with medical advice or death.

The most common form of complicated severe malaria among the studied 77 patients in Aden was cerebral malaria (25.9%), followed by renal failure, severe anemia, and hepatitis at 18.2%, 16.9%, and 14.3%, respectively (Table 1). 

The mean hemoglobin concentration showed a highly significant statistical difference in relation to the type of complications in severe malaria (p=0.00001); it was lowest in those with severe anemia (3.5 g/dL), increasing to 8.2 g/dL in cases of renal failure, to 9 and 9.1 g/dL respectively in hepatitis cases and in prostrated patients, and up to 11.4 and 11.6 g/dL in cerebral malaria and algid malaria patients, respectively (Table 1). 

Platelet and white blood cell (WBC) counts did not show significant statistical differences in relation to the type of complications in severe malaria (p=0.052 and p=0.095, respectively) (Table 1). 

The studied patients were followed until complete improvement and discharge, while 6 of them died, representing a case fatality rate of 7.8% (Figure 1). The mean age of deceased patients with severe malaria was statistically significantly older than the mean age of those who survived after severe malaria: 45.2 years versus 29.3 years, respectively (p=0.015). However, the mean values of the studied hematological parameters (hemoglobin, platelet, and WBC counts) were not significantly different among those who died or survived after severe complicated malaria (Table 2). 

Thirty-three patients from the studied 77 with severe complicated malaria had thrombocytopenia (platelet count of <150x109/L) at variable degrees; this rate represents 42.9% of all severe complicated malaria cases in this study. However, none of them developed bleeding. In these patients, the count was repeated by manual method. They were classified into 3 grades: grade 1 thrombocytopenia with counts of <150 to 75x109/L, grade 2 thrombocytopenia with counts of <75 to 50x109/L, and grade 3 thrombocytopenia with counts of <50x109/L. The studied cases of thrombocytopenia were classified as 57.6% in grade 1, 24.2% in grade 2, and 18.2% in grade 3 (Table 3). 

Three of the 6 deceased patients had grade 1 thrombocytopenia, 2 had grade 2, and 1 had grade 3 (Table 3). Five out of the 6 deceased patients in this study (83.3%) had cerebral malaria, while the sixth patient (16.7%) had renal failure (Table 4). Discussion This study demonstrated some hematological parameters in complicated severe malaria due to Plasmodium falciparum, which is the most common type of malaria infection in Yemen, accounting for 90% of malaria cases [5]. Cerebral malaria was the most common complication in severe malaria in adults, representing a quarter of the studied cases (25.9%). Several hypotheses have been proposed to explain the pathophysiology of cerebral malaria, but none have been completely satisfactory. Moreover, there is no association of cerebral malaria with altered hematological parameters [1]. This is similar to the findings of this study (Table 1), whereby mean hemoglobin, WBC, and platelet counts were not affected in cerebral malaria patients. Hemoglobin concentration showed lowest mean values reaching 3.5 g/dL in the studied patients with severe anemia. Plasmodium falciparum malaria is one of the most common causes of anemia. The other Plasmodium species rarely cause anemia because only select red cell populations (reticulocytes in the case of P. vivax and P. ovale and older cells in P. malariae) are invaded. Multiple mechanisms cause anemia in severe malaria, the most important being hemolysis of infected and uninfected red blood cells (RBCs), splenic sequestration of RBCs, dyserythropoiesis, and bone marrow suppression; such factors can culminate in the chronically low hemoglobin values observed in patients residing in holoendemic regions [8,9]. Studies found that anemia is also correlated with the severity of malaria infection [10]. In this study, the mean WBC count did not show a deviation from the normal reference range in all types of complicated severe malaria. This is similar to the results reported by Bashawri et al. [11] in Saudi Arabia. However, other studies showed that during severe P. falciparum infection there are changes in leukocyte proliferation and function. Thrombocytopenia was detected in 42.9% of the studied patients with complicated severe malaria. This is a common hematological alteration in malaria, which may be a result of peripheral platelet destruction and consumption. Studies showed that immune complexes generated by malarial antigens lead to sequestration of the injured platelets by macrophages in the spleen [12]. The present finding of thrombocytopenia is similar to that that reported by Banzal et al. [13] in Saudi Arabia (50.4%). In this study, thrombocytopenia was graded to look for the percentage of lower platelet counts; 18.2% of the studied patients showed a count of <50.0x109/L. In a study by Khan et al. [14], a quarter (26.8%) of the cases of P. falciparum malaria showed grade 3 thrombocytopenia with a count of <50.0x109/L. However, none of our patients bled, and all of them recovered from thrombocytopenia quickly during treatment. In regard to mortality associated with complicated severe malaria, neurological manifestations were the major determinant of morbidity and mortality in severe malaria cases in adults. All 6 of the deceased patients in this study suffered cardiopulmonary arrest and died. Postmortem autopsy is not a routine in Yemen; it is only done in legal cases. Five out of the 6 deceased patients in this study (83.3%) had cerebral malaria and the sixth (16.7%) had renal failure. However, hematological findings were not seriously deteriorated in the studied patients with cerebral malaria. This finding is similar to that reported by Mengistu and Diro in Ethiopia [15], as well as to the findings of Giha et al. [16] in Sudan, who reported mean hemoglobin levels to be higher in patients who died of severe malaria than in the survivors. 

## DISCUSSION

This study demonstrated some hematological parameters in complicated severe malaria due to Plasmodium falciparum, which is the most common type of malaria infection in Yemen, accounting for 90% of malaria cases [5]. 

Cerebral malaria was the most common complication in severe malaria in adults, representing a quarter of the studied cases (25.9%). Several hypotheses have been proposed to explain the pathophysiology of cerebral malaria, but none have been completely satisfactory. Moreover, there is no association of cerebral malaria with altered hematological parameters [1]. This is similar to the findings of this study (Table 1), whereby mean hemoglobin, WBC, and platelet counts were not affected in cerebral malaria patients. 

Hemoglobin concentration showed lowest mean values reaching 3.5 g/dL in the studied patients with severe anemia. Plasmodium falciparum malaria is one of the most common causes of anemia. The other Plasmodium species rarely cause anemia because only select red cell populations (reticulocytes in the case of P. vivax and P. ovale and older cells in P. malariae) are invaded. Multiple mechanisms cause anemia in severe malaria, the most important being hemolysis of infected and uninfected red blood cells (RBCs), splenic sequestration of RBCs, dyserythropoiesis, and bone marrow suppression; such factors can culminate in the chronically low hemoglobin values observed in patients residing in holoendemic regions [8,9]. 

Studies found that anemia is also correlated with the severity of malaria infection [10]. In this study, the mean WBC count did not show a deviation from the normal reference range in all types of complicated severe malaria. This is similar to the results reported by Bashawri et al. [11] in Saudi Arabia. However, other studies showed that during severe P. falciparum infection there are changes in leukocyte proliferation and function.

Thrombocytopenia was detected in 42.9% of the studied patients with complicated severe malaria. This is a common hematological alteration in malaria, which may be a result of peripheral platelet destruction and consumption. Studies showed that immune complexes generated by malarial antigens lead to sequestration of the injured platelets by macrophages in the spleen [12]. The present finding of thrombocytopenia is similar to that that reported by Banzal et al. [13] in Saudi Arabia (50.4%). 

In this study, thrombocytopenia was graded to look for the percentage of lower platelet counts; 18.2% of the studied patients showed a count of <50.0x109/L. In a study by Khan et al. [14], a quarter (26.8%) of the cases of P. falciparum malaria showed grade 3 thrombocytopenia with a count of <50.0x109/L. However, none of our patients bled, and all of them recovered from thrombocytopenia quickly during treatment. 

In regard to mortality associated with complicated severe malaria, neurological manifestations were the major determinant of morbidity and mortality in severe malaria cases in adults. All 6 of the deceased patients in this study suffered cardiopulmonary arrest and died. Postmortem autopsy is not a routine in Yemen; it is only done in legal cases. Five out of the 6 deceased patients in this study (83.3%) had cerebral malaria and the sixth (16.7%) had renal failure. However, hematological findings were not seriously deteriorated in the studied patients with cerebral malaria. This finding is similar to that reported by Mengistu and Diro in Ethiopia [15], as well as to the findings of Giha et al. [16] in Sudan, who reported mean hemoglobin levels to be higher in patients who died of severe malaria than in the survivors. 

## CONCLUSION

This study concluded that hematological changes are common complications encountered in any patient with severe malaria. Hemoglobin concentration is associated with significant changes in relation to the type of complications of severe malaria; however, it is not associated with the clinical outcome after severe malaria. The total WBC count cannot be used as a predictor for severity. Thrombocytopenia can implicate complications, but it is usually asymptomatic and platelet transfusions are generally not required because patients recover quickly. It is recommended that physicians should rely on the clinical presentation and complaints of patients with severe malaria and not hurry to conduct transfusion of blood or blood components based on the findings of hematological parameters alone (Table 5).

## CONFLICT OF INTEREST STATEMENT

The authors of this paper have no conflicts of interest, including specific financial interests, relationships, and/ or affiliations relevant to the subject matter or materials included.

## Figures and Tables

**Table 1:**
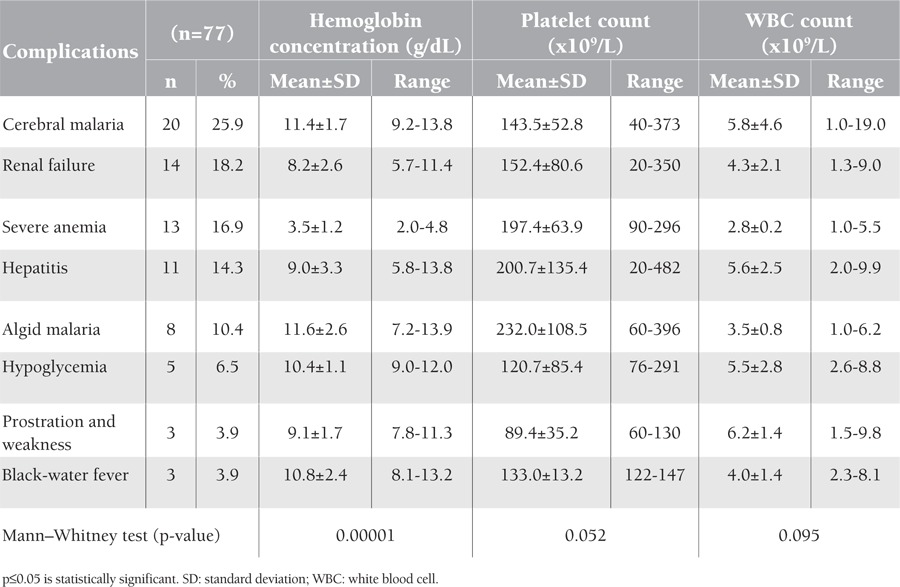
Studied hematological parameters by type of complications in severe malaria

**Table 2:**
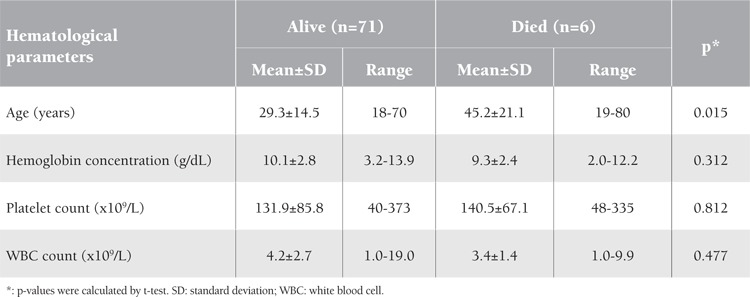
Age and studied hematological parameters of deceased versus surviving patients with complicated severe malaria

**Table 3:**
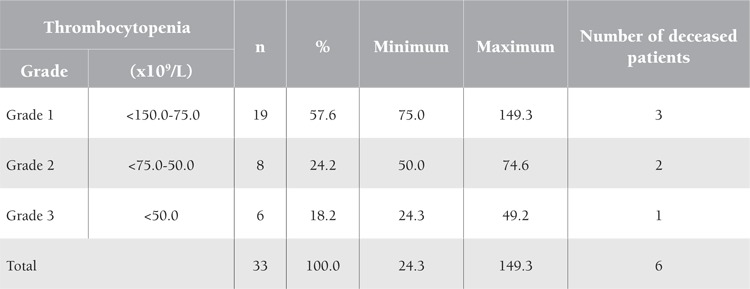
Grades of thrombocytopenia associated with complicated severe malaria in adult patients in Aden

**Table 4:**
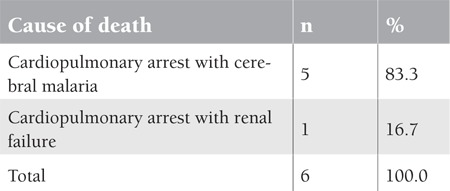
Causes of mortality in 6 adult patients with complicated severe malaria in Aden

**Table 5:**
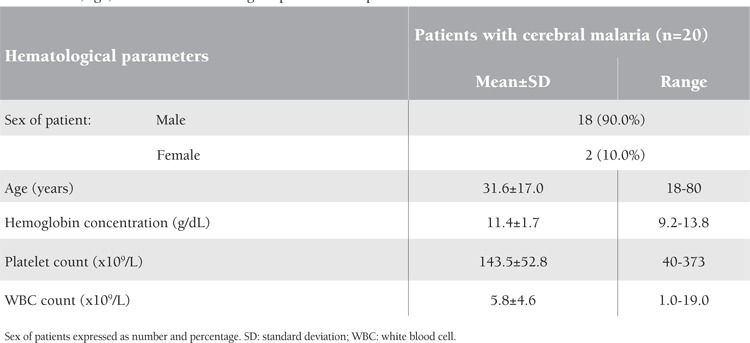
Sex, age, and studied hematological parameters in patients with cerebral malaria
